# Anxiety-like behavior and microglial activation in the amygdala after acute neuroinflammation induced by microbial neuraminidase

**DOI:** 10.1038/s41598-022-15617-5

**Published:** 2022-07-08

**Authors:** Ana León-Rodríguez, María del Mar Fernández-Arjona, Jesús M. Grondona, Carmen Pedraza, María D. López-Ávalos

**Affiliations:** 1 Departamento de Biología Celular, Genética y Fisiología, Facultad de Ciencias, Universidad de Málaga, Campus de Teatinos, 29071 Málaga, Spain; 2 Grupo de Investigación en Neuropsicofarmacología, Laboratorio de Medicina Regenerativa, Hospital Regional Universitario de Málaga, 29010 Málaga, Spain; 3 Departamento de Psicobiología y Metodología de Las Ciencias del Comportamiento, Facultad de Psicología Y Logopedia, Universidad de Málaga, Campus de Teatinos, 29071 Málaga, Spain; 4 Instituto de Investigación Biomédica de Málaga-IBIMA, Málaga, Spain

**Keywords:** Cognitive neuroscience, Emotion, Glial biology, Neuroimmunology, Social behaviour, Stress and resilience

## Abstract

Short-term behavioral alterations are associated with infection and aid the recovery from sickness. However, concerns have raised that sustained behavioral disturbances after acute neuroinflammation could relate to neurological diseases in the long run. We aimed to explore medium- and long-term behavioral disturbances after acute neuroinflammation in rats, using a model based on the intracerebroventricular administration of the enzyme neuraminidase (NA), which is part of some pathogenic bacteria and viruses. Neurological and behavioral assessments were performed 2 and 10 weeks after the injection of NA, and neuroinflammation was evaluated by gene expression and histology. No alterations were observed regarding basic neurological functions or locomotor capacity in NA-injected rats. However, they showed a reduction in unsupported rearing, and increased grooming and freezing behaviors, which indicate anxiety-like behavior. A principal component analysis including a larger set of parameters further supported such anxiety-like behavior. The anxiety profile was observed 2 weeks after NA-injection, but not after 10 weeks. Concomitantly, the amygdala presented increased number of microglial cells showing a morphologic bias towards an activated state. A similar but subtler tendency was observed in hypothalamic microglia located in the paraventricular nucleus. Also, in the hypothalamus the pattern recognition receptor toll-like receptor 4 (TLR4) was slightly overexpressed 2 weeks after NA injection. These results demonstrate that NA-induced neuroinflammation provokes anxiety-like behavior in the medium term, which disappears with time. Concurrent microgliosis in the amygdala could explain such behavior. Further experiments should aim to explore subtle but long-lasting alterations observed 10 weeks after NA injection, both in amygdala and hypothalamus, as well as mild behavioral changes.

## Introduction

Neuroinflammation is emerging as an underlying feature of a variety of neurological disturbances, ranging from mild behavioral alterations to more severe neurodegenerative diseases or even psychiatric disorders^1–6^. During neuroinflammation peripheral immune cells are recruited to the brain and resident immunocompetent cells (significantly microglia and astrocytes) activate, all of them contributing to the production of a plethora of inflammatory mediators including cytokines, chemokines and eicosanoids, among others^7^. All the events displayed during inflammation must be under a tight temporal control, including their resolution. Dysregulated neuroinflammatory episodes could result in the accumulation of inflammatory mediators with potentially neurotoxic effects, leading to behavioral alterations and neurological malfunction^8,9^. In humans, increased levels of pro-inflammatory cytokines have been observed in mental disorders such as schizophrenia^10^ and depression^11^. In these pathological situations, cytokines may be produced within the brain, mostly by activated microglia^12,13^ or by infiltrated immune cells, or can also be of peripheral origin. In fact, covid-19 pandemic has contributed additional evidences of how peripheral inflammation and the accompanying cytokine storm may impact memory, cognition and behavior, sometimes triggering or aggravating mental illnesses^14–16^. The so-called sickness behavior, described long ago, is a complex response that develops in animals during infections, with various symptoms such as malaise, loss of appetite, fever, sleepiness, tiredness, depression and anxiety. It has an adaptive value, as it allows preserving energy in favor of an effective immune response^17,18^, and a short-term time span, with most of the mentioned symptoms disappearing once inflammation and infection are solved. While short-term behavioral changes are well described, new evidences support the incidence of persistent behavioral symptoms associated with neuroinflammation^19–22^. So much so that anti-inflammatory agents have been proposed as treatment for psychiatric disorders^23–26^. Importantly, depression is emerging as a possible lingering consequence of inflammation associated to sickness, and cytokines are proposed to be essential players^27–29^. Therefore, knowing the connections between inflammation and behavioral disturbances and unveiling the underlying mechanisms are of priority importance.

Neuroinflammation can be triggered by a variety of stimuli, originating both central and peripherally, including pathogens, brain trauma or stroke, hypercaloric diets or psychosocial stress^5,30–32^. Among them, certain microbes (both viruses and bacteria) may enter the brain, where they trigger a pronounced inflammatory response^33,34^. Neuroinflammation induced by pathogens is frequently modeled by using bacterial components, the most widely used being the lipopolysaccharide (LPS)^35^. Viral components such as the viral RNA mimetic polyinosinic:polycytidylic acid are used to more specifically model a viral infection^36^. Neuraminidase is a sialidase present in the cell wall or the envelope of a wide range of bacteria and viruses, and notably in widespread viruses such as influenza, mumps and measles viruses; besides, some of these microorganisms are able to reach the CNS^37–42^. When delivered intracerebroventricularly (ICV) in rodents, NA provokes a sterile acute inflammatory process which recapitulates the hallmarks of neuroinflammation: infiltration of immune peripheral cells, activation of resident cells (microglia and astrocytes), increased levels of cytokines and chemokines, increased permeability of the blood brain barrier, and activation of the complement system^43–46^. The process peaks short after the injection of NA, and is virtually solved after 2–3 weeks. Thus, ICV-NA administration represents a suitable model of acute microbial-induced neuroinflammation, whose temporal course has been described previously^46^.

While some studies have addressed the impact of neurotropic viruses on acute neurological signs^33,34^, studies aimed at exploring the long-term neurological and behavioral consequences are scarce^47^. Here we pursued to explore if NA-induced neuroinflammation provokes behavioral or neurological sequelae at medium/long-term after NA injection. Because behavioral alterations during the acute phase of neuroinflammation have been extensively reported, evaluation at this acute phase was beyond the scope of this study. Also, signs of neuroinflammation were investigated, specifically in structures involved in stress responses and behavior such as the paraventricular nucleus of the hypothalamus or the amygdala.

## Methods

### Animals

Male Wistar rats of 8–10 weeks and about 300 g were acquired from Charles River Laboratories. The animals were housed in pairs with water and food available ad libitum, and a constant 12 h light/dark cycle, at 23 °C and 60% humidity.

All the experimental procedures were in accordance to the guidelines established by the European Union regulation (2010/63/EU), as well as the Spanish laws (RD 53/2013 and 178/2004, Law 32/2007 and 9/2003), and were also conducted in accordance with ARRIVE guidelines. All experimental protocols were approved by the ethics committee of Universidad de Malaga (Comité Etico de Experimentación de la Universidad de Málaga; reference 2012–0013). Manipulation of animals was done trying to minimize both the suffering and the number of animals used.

### Experimental design

**Figure 1:**
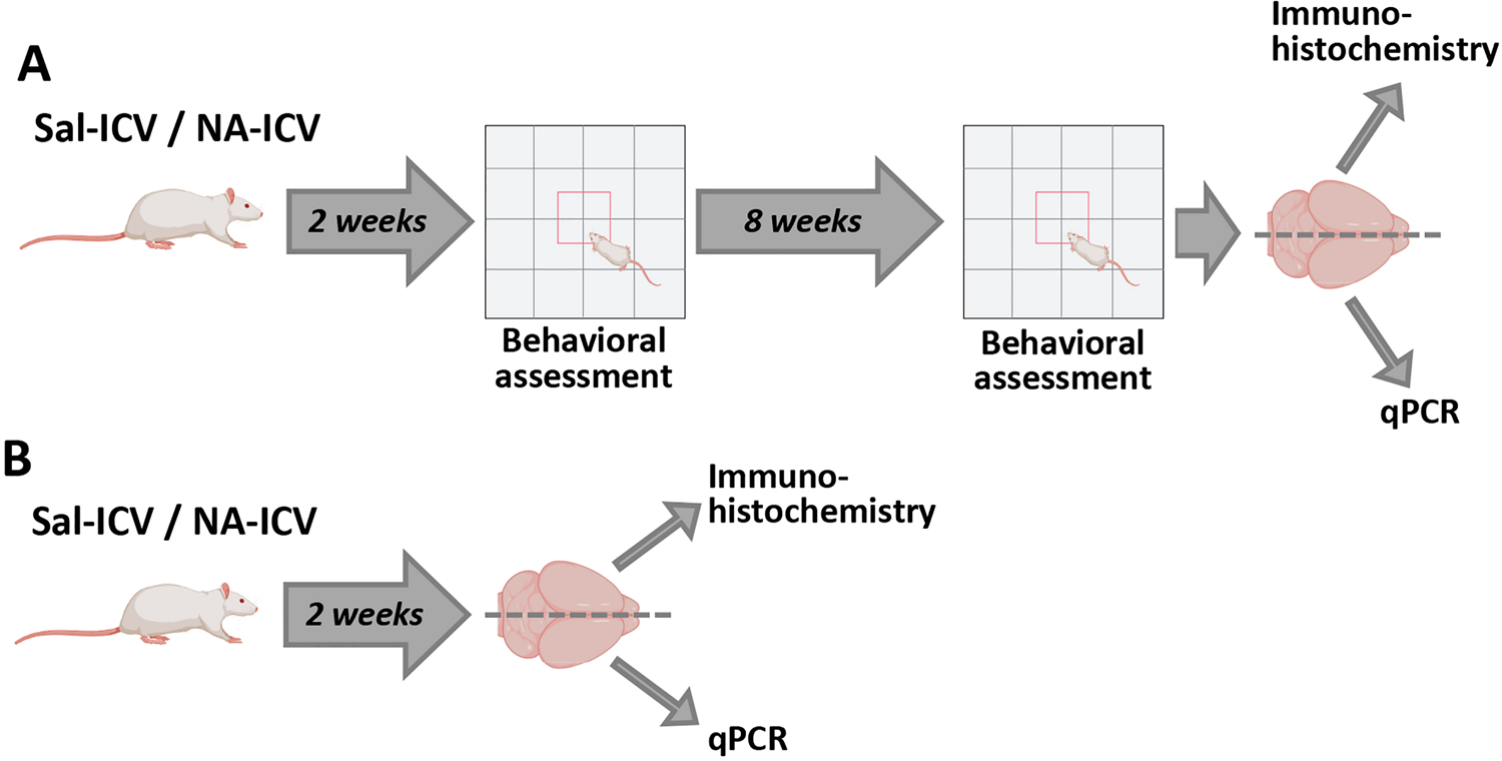
Schematic representation of the experimental design. (**A**) The rats were ICV-injected either with neuraminidase (NA) or with saline (Sal) as control. A behavioral assessment (consisting in open field test and a battery of neurological tests) was carried out 2 weeks after the ICV-injection, and later again at 10 weeks post-ICV. Then, the animals were sacrificed and their brains removed for the study of the inflammatory status by immunohistochemistry and qPCR. (**B**) Some animals were ICV-injected and sacrificed 2 weeks later, with the aim of obtaining brain tissue samples for studying the inflammatory situation at this time point.

Male rats (n = 22) were divided into two groups. One of them (n = 11) was injected intracerebroventricularly (ICV) with a single dose of the enzyme neuraminidase (NA) within the right lateral ventricle of the brain, to provoke an acute neuroinflammatory event^46^. The other group (n = 11) was ICV-injected in the same way with a sterile solution of 0.9% saline (control). After 2 weeks, all animals were subjected to a battery of neurological tests and a behavioral assessment by means of the open field (OF) test. Eight weeks later (10 weeks post-ICV injection) the rats underwent the same tests. After this second behavioral assessment the rats were sacrificed and their brains removed in order to check the inflammatory state (Fig. 1A). The brains were divided along the sagittal plane; one side was processed for histology and the other for qPCR.

An additional group of rats (n = 10) were ICV-injected (NA or saline) and sacrificed 2 weeks later, with the purpose of obtaining brain tissue to study the inflammatory situation at this time point (Fig. 1B).

### Intracerebroventricular injection

The rats were anesthetized with a solution of ketamine/xylazine (Sigma Aldrich) (80 and 12 mg/kg respectively) and placed in a stereotaxic frame. A small cut was done in the skin to expose the skull. Once the Bregma was located, a drill was used to pierce the bone in the coordinates corresponding to the right lateral ventricle: 0.5 mm posterior and 1.4 mm lateral from Bregma. A single dose of NA from *Clostridium perfringens* (Sigma-Aldrich, N3001) dissolved in 0.9% sterile saline solution (500 mU in 20 μL) was injected 3.5 mm below the dura mater. The solution was perfused using a pump, at a rate of 2 μL/min during 10 min. Control rats were injected with sterile saline solution. Once recovered from the anesthesia, rats were housed in the same original conditions.

### Neurological evaluation

The animals underwent a series of tests to determine potential neurological disturbances as a consequence of the ICV injection. The neurological functions tested were the sensorial reflexes (whisker touch, corneal reflex, head shaking, olfaction and auditory startle) and the limb reflexes and coordination (forelimb suspension, equilibrium test, equilibrium test with slope and surface righting reflexes^48,49^). They were performed few hours prior to running the OF test. Briefly, the tests were conducted as follows: (1) Whisker touch: from an area not covered by the animal’s visual field, whiskers were touched with the help of a swab; the expected response was the animal turn towards the origin of stimulus. (2) Corneal reflex: with a brush, the surface of the animal’s cornea was stimulated; the expected response was the animal to blink. (3) Head shaking: with the help of a tube, a puff of air was blown in the animal’s ear; the expected reaction was for the animal to shake its head. (4) Olfaction: the animal’s nose was approached with an ammonia swab; the expected reaction was the animal to step back from the source of the stimulus. (5) Auditory startle: close to the animal, a sudden loud noise was produced; the expected reaction was the animal to jump. (6) Forelimb suspension: the animal was suspended from a cable by both front legs; the expected reaction was the animal to grab the cable with the hind legs free and push itself up in less than ten seconds. (7) Equilibrium test: the tested animal was placed on a 2 cm wide and 30 cm long board, located 50 cm above the ground; the expected reaction was the animal to walk normally all along the board. (8) Equilibrium test with slope: the animal was placed looking downwards on an 30° inclined board; the expected reaction was to turn around and look upwards. (9) Surface righting reflexes: the animal was placed on a flat surface on its back; the expected reaction was to straight itself on its paws.

The outcome of these tests was rated using a three-point scale, where 2 was assigned when the animal reacted as expected and 0 when it did not. If the test was only partially well executed, the value assigned was 1. This neurological examination was evaluated by two different examiners who did not know which experimental group each animal tested belonged to.

### Open field test

The OF test was used to evaluate the locomotor capacity of the animals, as well as their explorative activity and various behaviors. This test was conducted 2 weeks and 10 weeks after the ICV injection of NA/saline. The test was carried out in a square box with a surface area of 90 × 90 cm and walls 45 cm high, made of grey plastic. The animal to be tested was placed in the center of the arena, from where it could start moving freely. The activity of the animal was recorded during 5 min with a video camera fixed 2 m above the box. The arena was cleaned with 70° alcohol each time a new animal run the test.

Using a video tracking system (Ethovision XT 7, Noldus, Wageningen, Netherlands), the following spatio-temporal parameters were assessed: (1) *total distance travelled* (cm), (2) *locomotion speed* (cm/s) (both of which inform about the locomotor capacity of the animals), (3) *time spent in the center* of the arena (s), and (4) *time spent in the periphery* of the arena (s).

Secondly, ethological patterns exhibited by the animals during the OF test (*freezing* behavior, *rearing*, *grooming*, *risk assessment*, and *head dipping*) were scored by two researchers blinded to the animal identity, with an inter-rater reliability measure over 80% using the software Raton Time 1.0 (Fixma S.L., Valencia, Spain). The behaviors selected to be evaluated^50^ were: (1) *rearing* and (2) *rearing with support*, which refer to the time that the animal spends exploring the environment standing up on its hind legs with no support (*rearing*) or supporting itself against the walls of the box (*rearing with support*). (3) *Freezing*, that is, when the animal freezes and the only muscles moving are those involved in breathing. (4) *Grooming*, or repetitive episodes of self-directed and sequentially patterned behaviors of hygiene and self-care. For each of these behaviors, the duration, the frequency (number of episodes in 5 min), the latency (defined as the time from the rat being placed in the open field to the first bout of each behavior lasting longer than 2 s), and the time/frequency ratio were calculated.

### Sacrifice and tissue sampling

The rats were anesthetized with the same solution of ketamine/xylazine used for the ICV-injection. Then they were perfused transcardially with a solution of 0.9% NaCl and 10 U/mL heparin, in order to clear the blood from tissues. The brain was then removed and divided by the midline, in order to use the same brain for both quantitative PCR and histological studies. The half of the brain intended for histology was placed in 4% paraformaldehyde fixative solution overnight at 4 °C. The other half was dissected to obtain the hypothalamus, which was immediately frozen and stored at − 80 °C until processed for RNA extraction.

### RNA isolation

The hypothalamic tissue was homogenized mechanically in RNAzol (0.5 mL/50 mg of tissue; Molecular Research Center Inc.). Total RNA was extracted following manufacturer’s indications. To dissolve the RNA pellet obtained, 30 μL of RNAse-free water were added and mixed with a vortex. RNA samples were stored at − 80 °C. Quantification of the RNA extracted was done in a Nanodrop device. The purity of RNA samples was considered acceptable if the ratio of absorbances at 260 and 280 nm (A260/A280) was approximately 1.8.

### Reverse transcription

The synthesis of cDNA from RNA isolated from the hypothalamus was performed by a reverse transcription reaction. RNA samples were diluted with RNAse-free water to obtain the same concentration of RNA in all of them (1500 ng in 8 μL). Each reaction tube included 8 μL of sample containing 1500 ng of RNA and 2 μL of the PrimeScript RT Master Mix reaction mixture (Takara, RR036A) in a final volume of 10 μL. The reverse transcription reaction consisted in 15 min at 37 °C, followed by 5 min at 85 °C for reverse transcriptase inactivation. The 10 μL cDNA sample was then diluted with 90 μL of water to obtain a cDNA concentration equivalent to 15 ng of cDNA equivalent/μL. cDNA samples were stored at − 20 °C.

### Quantitative PCR (qPCR)

The sequences of the genes of interest were searched in the Genbank NCBI Reference Sequence. Primers were designed using the Primer Blast program (https://blast.ncbi.nlm.nih.gov), and are listed in Supplementary material (Table S1). The level of target mRNA in the samples was quantified using a real-time PCR based on SYBR Green I fluorescence dye. For this purpose, the hot start reaction mix FastStart Essential DNA Green Master (Roche: 06402712001) was used. To obtain a final reaction volume of 20 μL/tube, the following reagents were added in 96-well PCR plate: 10 μL master mix, 2 μL of both primers (at a concentration of 0.4 μM each), and 8 μL of cDNA (120 ng of cDNA/μL). The PCR reaction was carried out in a LightCycler® 96 equipment (Roche). Amplification curves, dissociation curves and quantification cycles (Cq) were obtained for of each target gene. All data was processed using the software of the LightCycler® 96 equipment.

Prior to running the qPCR with the samples, the PCR efficiency (E) was estimated, by doing the amplification in serial dilutions of the cDNA samples. E was obtained from the equation^51^ E = 10 ^[− 1/slope]^ – 1. The level of expression of the target genes was relativized to the expression of a reference gene, in this case glyceraldehyde 3-phosphate dehydrogenase (GAPDH). The expression of each target gene relative to the expression of GAPDH was calculated as follows:

$${\text{Gene expression }}\left( {{\text{relative to GAPDH}}} \right)\, = \,\left( {{\text{E}}_{{target}} } \right)^{{\Delta {\text{CP}}target}} /\left( {{\text{E}}_{{GAPDH}} } \right)^{{\Delta {\text{CP}}GAPDH}} .$$

where E is the efficiency of the PCR reaction for each of the genes (the target gene and the reference gene GAPDH) and ΔCP is the difference between the Cq values of the control sample and the experimental sample, for both target and reference genes.

### Immunohistochemistry

The fixed brains were sectioned in the coronal plane using a vibratome, obtaining sections of 40 μm thickness. The sections selected for the immunohistochemistry were those including the basolateral amygdala and the paraventricular nucleus (PVN) of the hypothalamus (between − 1.5 mm and − 2.0 mm from Bregma approximately).

With the aim of studying cell populations involved in the neuroinflammatory process, three different antibodies were used: anti-ionized calcium-binding adapter molecule 1 (IBA1, to identify microglia; although also labels peripheral macrophages), anti-glial fibrillary acidic protein (GFAP, a marker of astrocytes) and galectin 3 (Gal3, to identify infiltrated peripheral cells).

Free floating sections were washed with PBS and incubated in PBT solution (0.3% bovine serum albumin and 0.3% Triton X-100 in PBS pH 7.3) to block the non-specific binding sites. Primary antibodies (rabbit polyclonal anti-IBA1 1:1000, Wako; rabbit polyclonal anti-GFAP 1:2000, Sigma-Aldrich) were incubated at 4 C overnight. After PBS washes, the sections were incubated with a biotinylated secondary antibody (goat anti-rabbit 1:1000; Pierce) during 1.5 h. To detect the secondary biotinylated antibodies, an amplification system based on the avidin–biotin-complex (ABC 1:250; Thermo Fisher Scientific) was used afterwards during 45 min. The peroxidase reaction was developed incubating the sections during 10 min with a solution containing 0.05% diaminobenzidine and 0.03% hydrogen peroxide in PBS. After washes with PBS, the stained sections were mounted onto gelatin-coated slides, air dried, and the following day counterstained with 0.1% toluidine blue solution during 10 min. Afterwards, the sections were dehydrated in graded ethanol, cleared in xylene and covered with a coverslip and Eukitt mounting medium for microscopy.

Peripheral cell infiltration was identified by immunofluorescence using as primary antibody goat anti-Gal3 (1:500; R&D Systems). The secondary antibody was a donkey anti-goat Alexa 568 (1:1000; Molecular Probes). Finally, the sections were mounted onto gelatin-coated slides, and coverslipped using anti-fading agent Mowiol 4-88 (Calbiochem/EMD Chemicals).

The negative control for immunohistochemistry consisted in by-passing the primary antibodies.

### Image acquisition

Images of DAB-stained IBA1-positive or GFAP-positive cells were obtained with an Olympus VS120 scanner microscope. With the purpose of cell counting, images were acquired with the UPLSAPO 40 × objective. The areas scanned were those corresponding to the PVN and the amygdala.

Images for the morphometric analysis of microglia were taken with the same scanner microscope, but using the UPLSAPO 60 × oil immersion objective, which yields high-resolution images (pixel size of 0.013 µm^2^; TIFF format). In this case, 20 images of 1 μm thickness were obtained by means of the multi-plane virtual-Z mode, spanning 20 μm depth within the tissue section. Between 2 and 4 images of the PVN/amygdala were taken from each animal. These images were later processed for the morphometric analysis of microglial cells.

Lastly, images of Gal3 immunofluorescence were captured with a 10 × objective in an inverted confocal microscope LEICA SP5 II, overlapping fluorescence with the bright field; several planes in the z-axis were captured and stacked in a single projected image.

### Cell counts

Using the images acquired with the purpose of cell counts, the number of cells in each of the areas studied was manually recorded using the software plugin Cell Counter for ImageJ, and the results were represented as the number of cells/mm^2^. Between 2 and 4 images (exceptionally 1 due to tissue section damages) from each of the structures (PVN/amygdala) were counted for each animal.

### Image processing and morphometric analysis

In those images obtained for the morphological analysis of microglia, individual microglial cells were selected and cropped, based on the following criteria: (1) random selection of the cells (in the PVN, starting in the wall of the third ventricle and moving towards the parenchyma; in the amygdala, from the dorsal part and moving to the basal area); (2) cells that were separated, not overlapping with any other nearby cell; (3) complete soma and branches visible in the sample. From each animal, about 10 cells were randomly selected. In the case of the amygdala, the total number of cells analyzed was n = 50–57 per experimental group, sampled from n = 5–6 animals. In the PVN, the number of cells analyzed per experimental group was n = 40–60, sampled from n = 4–6 animals.

The morphometric analysis was then performed using the free software FIJI (accessible from http://fiji.sc/Fiji). The cropped images of individual microglial cells were processed as follows: (1) Colors were split in three channels (red, blue and green); the green channel was used hereafter, as the brown label of IBA1 staining was enhanced in this channel. (2) The green-channel image was processed into a binary image (a threshold value in the grayscale was previously determined and applied to all images). (3) The binary image was manually edited in order to reproduce as faithful as possible the original color image. (4) From the resulting image, two different images were obtained: a filled image of the cell´s shape, and an outlined image showing only the profile of the cell. Both of them were needed for measuring different morphological parameters, a process that was performed with the free software plugin FracLac for ImageJ (Karperien A., FracLac for ImageJ1. 1999–2013; available at the ImageJ website, National Institutes of Health)^52^. Several parameters were measured: cell area, cell perimeter, cell circularity, fractal dimension, roughness, lacunarity, density, convex hull (CH) span ratio (CHSR), convex hull area (CHA), CH perimeter, CH circularity, maximum span across the convex hull (MSACH), the ratio CH radii, the mean radius and the bounding circle diameter (BCD)^43^. A graphical explanation of these parameters is available in the Supplementary material (Fig. S1).

### Statistical analysis

Statistical procedures were carried out with software SPSS Statistics® 24 (IBM®) and GraphPad Prism 9. Results are presented as the mean ± standard error of the mean (SEM). A *p* value ≤ 0.05 was established for the differences between means to be considered statistically significant. Extreme upper or lower values were identified as outliers in box plots and discarded from the statistical analysis. The normal distribution of the different data sets was checked with a Shapiro–Wilk test. The homoscedasticity of the distributions was checked with a Levene’s test for equality of variances. If both, the equality of variances and the normal distribution of the data, were accepted, the data set was considered as parametric; otherwise, the set of data was considered as non-parametric.

### Statistical analysis of behavioral tests data

The duration, latency, frequency and time/frequency ratio of the different behaviors exhibited by the animals during the OF test, and the velocity and the total distanced walked by the rats during the 5 min recorded in the OF area, were analyzed using a Student's *t*-test (*t*). Welch's *t*-test (*t*_*w*_) was performed when the two compared groups had unequal variances.

The non-parametric data set obtained from the neurological test was analyzed using Mann–Whitney U test.

With the data obtained in the OF test, a principal component analysis (PCA) with varimax rotation was performed, in order to bring out relationships between the different behavioral parameters, previously analyzed in the Student's *t*-test. With the PCA test it is possible to reduce the number of variables and to transform them into a new group of variables, which have the following characteristics: (1) new variables are a linear combination of the previous ones, (2) are uncorrelated among them, and (3) can explain a considerable percentage of the variance. Precisely, in order to accept the results from the PCA, the new variables (or Factors) should explain more than 60% of the variance.

The correlation matrix of the whole sample of animals (n = 22 vehicle and NA treated animals, both assessed at 2 or 10 weeks after ICV administration) was used for the analysis and tested for sampling adequacy by the Bartlett sphericity and the Kaiser-Meyer-Olking (KMO) tests. The resulting factors with the eigenvalue > 1 were selected. “Factor loading” (i.e. the contribution of each variable to a factor) was considered significant when it was > 0.05.

Subsequently, as the factor scores are a representation of the relative contribution of each loading pattern, Student's *t*-test (*t*) was applied in order to determine if there were differences between animals treated with NA and saline controls, both at 2 and 10 weeks after the ICV injection.

### Statistical analysis of data related to brain inflammation and microglial activation

Student's *t*-test (*t*) was used to evaluate differences between NA-injected rats and saline controls in parameters related to inflammation, that is, data sets from qPCR, from IBA1 and GFAP cell counts, and from microglial morphological parameters.

### Ethics approval

This study was performed in line with the guidelines established by the European Union regulation (2010/63/EU), as well as the Spanish laws (RD 53/2013 and 178/2004, Law 32/2007 and 9/2003). All experimental protocols were approved by the ethics committee of Universidad de Malaga (Comite Etico de Experimentacion de la Universidad de Malaga; reference 2012–0013). All experimental procedures were also conducted in accordance with ARRIVE guidelines.

## Results and statistical analyses

### Neurological consequences of NA-induced neuroinflammation

The concurrence of neuroinflammation with neurological alterations has been extensively reported. The ICV injection of the enzyme neuraminidase (NA) represents a model of acute neuroinflammation, which is solved in few days. Thus, 2 weeks after NA administration signs of inflammation are already scarce^46^. However, unpublished results by our group revealed long-term sequelae, particularly in microglia and astrocytes, which suggests a sustained mild neuroinflammation state. Therefore, we aimed to investigate if NA-induced neuroinflammation might also result in behavioral or neurological disturbances at medium (2 weeks) and long (10 weeks) term.

Neuraminidase was injected ICV in adult rats, and the animals were evaluated at two time points after the injection: 2 weeks later, when the acute phase of inflammation is resolved but some microglial and astrocytic activation remains^46^, and 10 weeks later, a time long enough for inflammation to be completely cured (Fig. 1).

First, in order to study the effects of NA administration on sensorimotor or exploratory functions, a battery of neurological tests was conducted in NA-injected and saline-injected (control) rats. Mann–Whitney U test showed no significant differences between treatments neither at 2 weeks nor at 10 weeks after the ICV injection (Supplementary material, Table S2). Therefore, NA injection did not result in major neurological disturbances, at least from 2 weeks post-injection onwards. However, we cannot rule out neurological damages in shorter times.

### Anxiety-like behavior after NA-induced neuroinflammation

**Figure 2:**
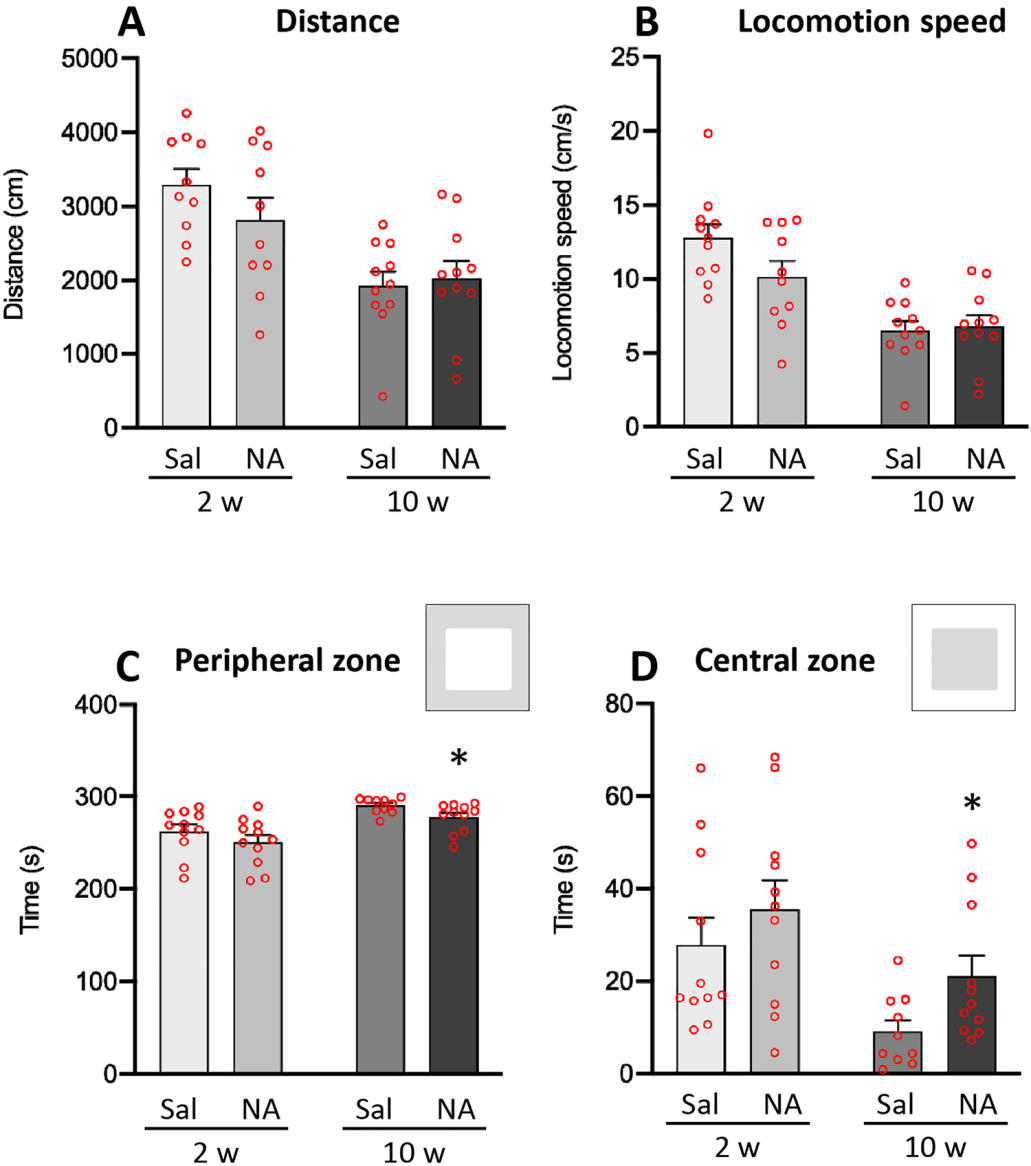
Parameters related to the locomotor capacity and area preference in the open field test. Animals were ICV-injected either with saline (Sal) or with neuraminidase (NA), and subjected to the open field test 2 weeks (2w) or 10 weeks (10w) after the ICV injection. The mean values ± SEM (n = 11 for both NA and Sal) of the *distance travelled* by the animals (**A**), the *locomotion speed* (**B**), the time spent in the *peripheral zone* (**C**) and the time spent in the *central zone* (**D**) are represented in histograms. Student’s t-test was performed in order to check the influence of treatment (Sal versus NA) at both assessments. *p < 0.05.

Next, behavioral assessment of the animals was carried out using the OF test. For the evaluation of locomotor capacity, the velocity and the distance that the animals walked during the 5 min duration of the test were measured. Statistical analysis revealed that within 2 weeks of NA administration there were not significant differences between treatments in the *distance travelled* (t = 1.274, df = 18, *p* = 0.219; Fig. 2A) or in the *locomotion speed* (t = 1.857, df = 19, *p* = 0.079; Fig. 2B). Similarly, no differences were observed 10 weeks after NA administration neither in the *distance travelled* (t = − 0.345, df = 20, *p* = 0.734; Fig. 2A) nor in the *locomotion speed* (t = − 0.284, df = 20, *p* = 0.779; Fig. 2B). These data seem to indicate that motor skills were not affected after NA administration, at least from 2 weeks onwards.

In relation to the time the animals spent in each of the zones established in the OF arena (Fig. 2), no differences were observed in the time spent in any of the compartments (peripheral and central zone) at 2 weeks of NA administration (for peripheral zone: t = 1.073, df = 20, *p* = 0.296; Fig. 2C; for central zone: t = − 0.898, df = 20, *p* = 0.380; Fig. 2D). However, 10 weeks after the injection animals treated with NA spent less time in the peripheral zone (t = 2.307, df = 15.564, *p* = 0.035; Fig. 2C), and consequently more time in the central zone (t = − 2.338, df = 15.355, *p* = 0.033; Fig. 2D).

**Figure 3:**
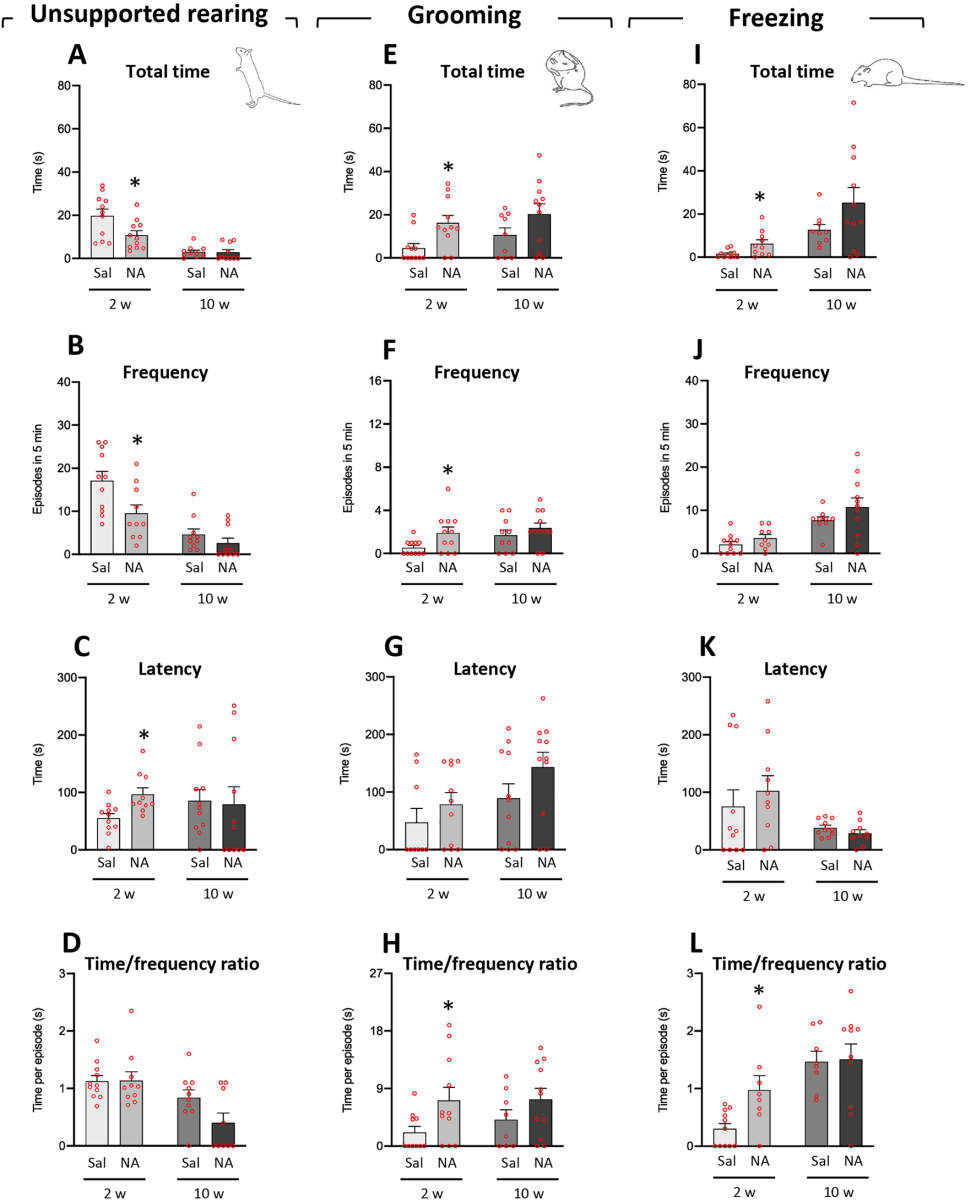
Parameters related to unsupported rearing, grooming, and freezing behaviors analyzed in the open field test. Rats were ICV-injected either with saline (Sal) or with neuraminidase (NA), and subjected to the open field test 2 weeks (2w) or 10 weeks (10w) after the ICV injection. Several parameters (*total time, frequency, latency* of the first episode, and the *time/frequency ratio*) related to the behaviors unsupported rearing (**A**–**D**), grooming (**E**–**H**) and freezing (**I**–**L**) were studied, and the mean values ± SEM (n = 11 for both NA and Sal) are represented in histograms. Student’s t-test were performed in order to check the influence of treatment (Sal versus NA) at both assessments. *p < 0.05.

On the other hand, based on an approach that relies on monitoring freely behavior of rats^53^ the ethological analysis of behavior exhibited by animals in the OF test was carried out. The following behavioral parameters were analyzed (including time, frequency, latency to the first episode and time/frequency ratio of each behavior) at both times points (2 and 10 weeks after NA administration): *unsupported rearing*, *supported rearing*, *grooming* and *freezing*. Regarding *unsupported rearing* (Fig. 3A–D), 2 weeks after ICV injections the animals treated with NA showed a reduction in the time (t = 2.445, df = 20, *p* = 0.023; Fig. 3A) and the frequency (t = 2.562, df = 19, *p* = 0.019; Fig. 3B), while an increase in the latency of the first episode (t = − 3.033, df = 16.675, *p* = 0.008; Fig. 3C); the ratio time/frequency of *unsupported rearing* was unaffected (t = − 0.063, df = 15.428, *p* = 0.950); Fig. 3D). No differences were found for any of these parameters at the evaluation carried out after 10 weeks (Fig. 3A–D). However, in relation to *rearing with support* (Supplementary material, Fig. S2) no statistically significant differences were observed between treatments in any of the parameters examined (for time: t = 1.904, df = 20, *p* = 0.071; for frequency: t = 0.706, df = 19, *p* = 0.489; for latency: t = − 0.386, df = 20, *p* = 0.704; for time/frequency ratio: t = 1.572, df = 19, *p* = 0.132).

Regarding the *grooming* behavior (Fig. 3E–H), data revealed that NA treated animals presented increased time (t = − 2.882, df = 16.647, *p* = 0.011; Fig. 3E), frequency (t = − 2.331, df = 12.815, *p* = 0.037; Fig. 3F), and ratio time/frequency (t = − 2.211, df = 14.036, *p* = 0.044; Fig. 3H) in the evaluation performed 2 weeks after the ICV injection, while not after 10 weeks; no statistically significant difference was found in the latency to the first grooming episode (t = − 0.983, df = 16.721, *p* = 0.340; Fig. 3G).

Moreover, as to *freezing* behavior (Fig. 3I–L), animals injected with NA showed higher values of the parameters time (t = − 2.471, df = 10.589, *p* = 0.032; Fig. 3I) and time/frequency ratio (t = − 2.511, df = 8.943, *p* = 0.033; Fig. 3L), while no differences for frequency (t = − 1.375, df = 18, *p* = 0.186; Fig. 3J) or latency (t = − 0.702, df = 18.945, *p* = 0.492; Fig. 3K).

No statistical differences were observed between NA and saline injected animals in any of these parameters (*unsupported rearing*, *supported rearing*, *grooming* and *freezing*) when examined at 10 weeks post-injection.

Thus, these results indicate that after 2 weeks of the ICV-injection of NA the rats show an enhancement in *grooming* and *freezing* behaviors, while a reduction in *unsupported rearing*. These differences are no longer observed 10 weeks after the injection.

**Figure 4:**
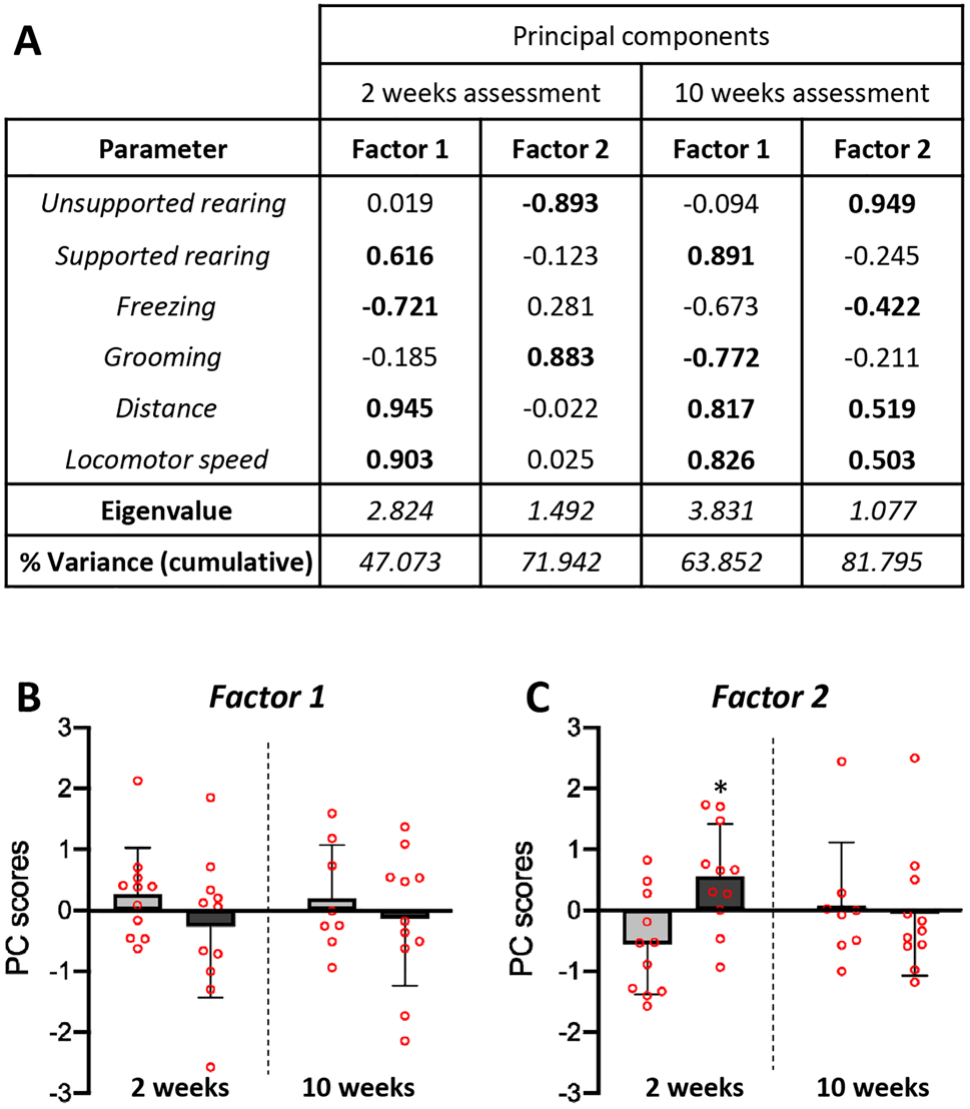
Principal component analysis (PCA) carried out with behavioral parameters obtained in the open field test. The PCA was carried out with the data obtained in the behavioral assessment performed at 2 weeks (2w) post-ICV injection as well as 10 weeks (10w) post-ICV injection. For the data obtained in the 2 weeks assessment KMO = 0.506, χ^2^ = 83.839, and P < 0.001. For the data from 10 weeks assessment KMO = 0.758, χ^2^ = 128.546, and P < 0.001. The PC scores for each of the parameters used in the PCA and the factors resulting (Eigenvalue > 1) from the analysis are presented (**A**). Interpretable factor loadings (> 0.50) are highlighted in bold, being positively or inversely correlated with each factor. The parameters’ scores in each factor suggest to relate Factor 1 to locomotor capacity, and Factor 2 to the animal’s emotional state. Representation with histograms of the PC scores for Factor 1 (**B**) and Factor 2 (**C**) obtained in the PCA at both evaluation times. Animals ICV-injected with NA showed the highest score for Factor 2 after 2 weeks of the ICV. In order to reveal any significant difference between treatments, a Student’s *t*-test was conducted. The bars represent the mean value ± SEM. Significant differences are shown with asterisk: *p < 0.05.

With the aim of determining how all of the previous parameters relate to each other and if their relationship could reveal a particular behavior, a principal component analysis (PCA) with a variance-maximizing rotation (varimax) was conducted. A two-component solution was uncovered by the PCA at both evaluation times, 2 and 10 weeks after the ICV treatment (Fig. 4A). In the OF test performed 2 weeks after the ICV injection the two components revealed by PCA accounted for 71.942% of the total variance. Strong positive correlations were found among *locomotor speed*, *distance* and *rearing with support,* and negative correlations with *freezing* behavior, in Factor 1 (suggesting that Factor 1 was related to the locomotor capacity of the animal). In Factor 2, a strong positive correlation was uncovered with *grooming*, as well as a strong negative correlation with *unsupported rearing* (thus Factor 2 was possibly related to the animal's emotional state; a high score may indicate increased anxiety). In order to study the possible effect of NA-treatment on Factor 1 and Factor 2, factor scores were calculated, and a Student’s *t*-test was used to compare NA and saline experimental groups. Regarding Factor 2, 2 weeks after the ICV injection those animals treated with NA exhibited a greater state of anxiety than control animals (t = − 3.124, df = 20, *p* = 0.005; Fig. 4C), whereas there was no significant difference between treatments in locomotor capacity (Factor 1; Fig. 4B).

As to the behavioral assessment carried out at 10 weeks post-injection, the two-component solution uncovered with the data obtained (accounting for the 81.795% of the total variance; Fig. 4A) was interpreted with the same possible factors as in the 2 weeks assessment (Factor 1 related to locomotion and Factor 2 related to anxiety). However, no significant differences were found between the two experimental groups neither on the locomotor capacity (Factor 1, Fig. 4B) nor the anxiety state (Factor 2, Fig. 4C).

Therefore, the PCA unveiled a Factor 1 related to locomotion and a Factor 2 related to anxiety, the latter being significantly increased 2 weeks after the treatment with NA, but not in the later evaluation performed 10 weeks after the treatment.

### Evidences of mild inflammation 2 weeks after the ICV injection of NA

**Figure 5:**
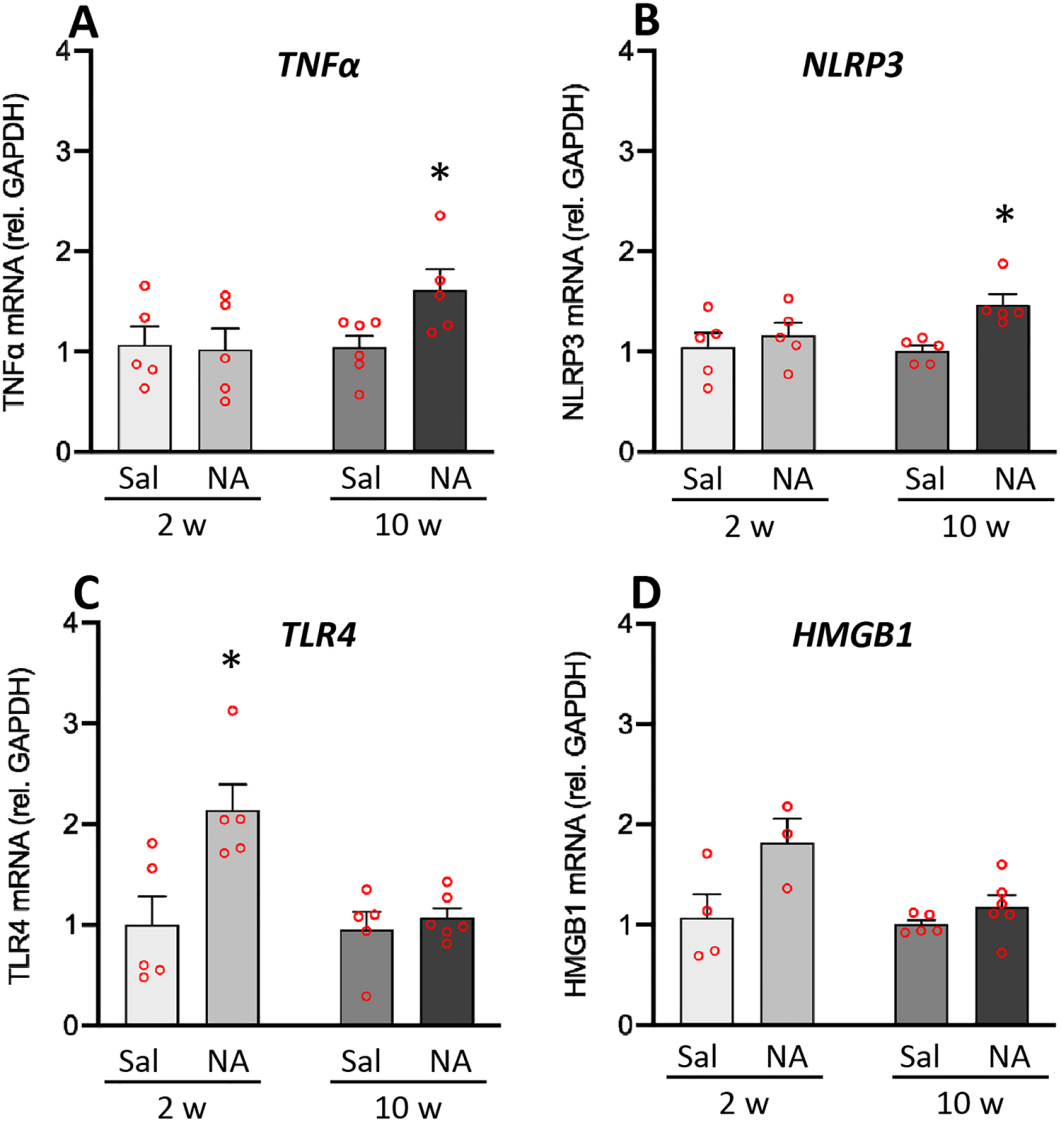
mRNA levels of genes related to neuroinflammation 2 and 10 weeks after the ICV administration of neuraminidase. Animals ICV-injected either with saline (Sal) or with neuraminidase (NA) were sacrificed 2 weeks (2w) or 10 weeks (10w) after the ICV. Gene expression was measured by qPCR in hypothalamic tissue samples. The mRNA levels of the inflammatory cytokine TNFα (**A**), the inflammasome protein NLRP3 (**B**), the receptor TLR4 (**C**) and the alarmin HMGB1 (**D**) were quantified relative to GAPDH expression. Student’s t-test was performed in order to check the influence of treatment (Sal versus NA) on the expression levels of these genes at both time points. The bars in histograms represent the mean value ± SEM of n = 5–6 animals per experimental group. *p < 0.05.

In order to study the inflammatory profile of the animals and its potential association to the behavioral results observed, first the mRNA levels of various genes related to inflammation were measured by qPCR in the hypothalamus (Fig. 5). This brain region was chosen because it is related to the stress response and is far away from the surgical damage provoked by the ICV injection. The genes studied were the pro-inflammatory cytokine tumor necrosis factor alfa (TNFα), the Toll-like receptor type 4 (TLR4), the alarmin high mobility group box 1 (HMGB1) and the inflammasome protein NLR family pyring domain containing 3 (NLRP3).

Within 2 weeks after the ICV injections no significant differences were found in the expression of the cytokine TNFα (t = 0.161, df = 8, *p* = 0.876; Fig. 5A). Nevertheless, after 10 weeks of the ICV, animals injected with NA showed an increased TNFα expression in comparison with the ones injected with vehicle (t = − 2.5, df = 9, *p* = 0.034; Fig. 5A). A similar result was observed regarding the expression of the inflammasome protein NLRP3, that is, similar mRNA levels 2 weeks after the ICV injection (t = − 0.614, df = 8, *p* = 0.556; Fig. 5B), while increased mRNA levels in NA treated rats 10 weeks after the injection (t = − 3.897, df = 6.184, *p* = 0.008; Fig. 5B).

Also, a significant increase in the expression of the receptor TLR4 was found 2 weeks after the ICV injection of NA (t = − 2.977, df = 8, *p* = 0.018; Fig. 5C), a difference that was no longer observed after 10 weeks (t = − 0.578, df = 6.164, *p* = 0.584; Fig. 5C).

Finally, regarding the expression of the alarmin HMGB1, no significant differences were found between NA and saline injected rats, neither 2 weeks nor 10 weeks after the ICV injection (t = − 2.168, df = 5, *p* = 0.082; t = − 1.370, df = 6.302, *p* = 0.218, respectively; Fig. 5D). A similar tendency to that seen in the expression of TLR4 was observed (increased mRNA levels in NA treated rats 2 weeks post-injection), however it was not statistically endorsed.

**Figure 6:**
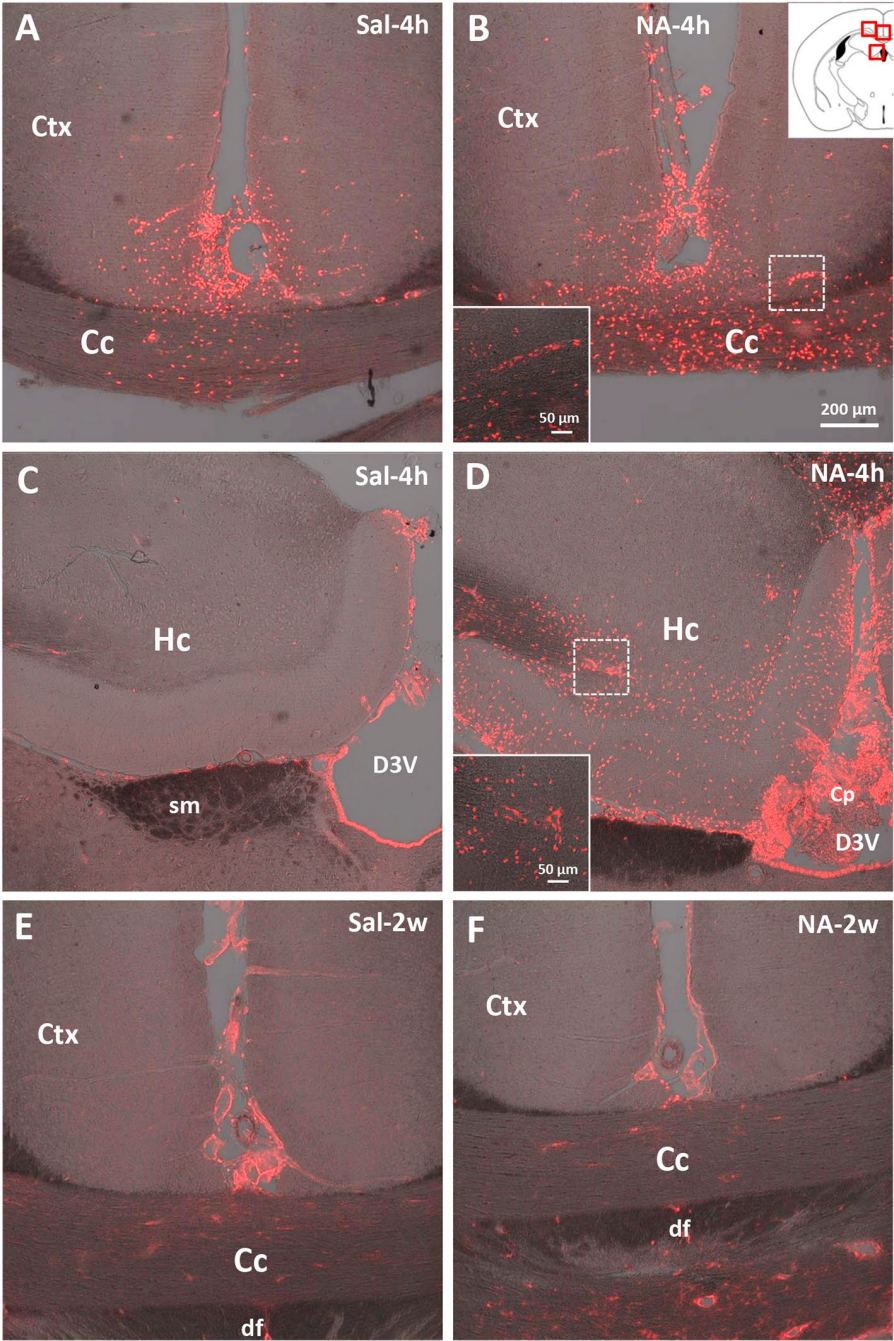
Infiltration of peripheral cells revealed by Gal3 immunostaining. Both animal groups, ICV-injected either with saline (Sal) or with neuraminidase (NA), were sacrificed 4 h (4 h) or 2 weeks (2w) after the ICV. Their brains were removed and processed for Gal3 immunostaining. Rats sacrificed 4 h post-injection (**A**–**D**) belong to other study, and were used here as a positive control for cell infiltration. The sections immunostained correspond to levels close to the ICV-injection site (see scheme at the top right, where the photographed areas are squared in red). Gal3-positive cells (stained in red) appear in rats sacrificed 4 h after the injection (**A**–**D**), and are noticeably more abundant in those ICV-injected with NA (**B**,**D**), revealing infiltration of peripheral cells shortly after the injection. Infiltrated cells are mainly located in the cerebral cortex (Ctx), the corpus callosum (Cc) as well as in the hippocampus (Hc) and the choroid plexus (Cp) located in the dorsal third ventricle (D3V), all close to the injection site. No infiltrating cells are seen after 2 weeks of the ICV injection neither with saline (**E**) nor with NA (**F**). Images in (**A**–**F**) are a z-stack projection of several confocal planes. Insets in (**B**) and (**D**) are higher magnifications of the regions squared with a broken line in each respective image, and correspond to a single confocal plane. *sm* stria medullaris thalamus, *df* dorsal fornix.

On the other hand, peripheral cell infiltration during neuroinflammation was assessed by immunostaining with anti-galectin 3 (Gal3) antibody (Fig. 6). Gal3 is an endogenous lectin expressed by myeloid cells including monocytes and neutrophils^54^. After the ICV injection of NA, cellular infiltration peaks between 4 and 24 h later^46^. Here we confirmed that 2 weeks after ICV injection Gal3-positive infiltrated cells were virtually absent (Fig. 6E,F) as well as 10 weeks later (not shown). As a positive control, sections from animals sacrificed 4 h after the injection (from other experiments not presented here) were used. Gal3 immunostaining showed substantial cell infiltration in areas close to the ICV injection site, such as the cortex, corpus callosum, and hippocampus , which was much milder in saline (Fig. 6A,C) than in NA-injected animals (Fig. 6B,D). The hypothalamus was virtually devoid of infiltrated cells (not shown), as expected because of its location further from the injection site. Thus, although infiltration of immune cells to the CNS is a relevant event in the hours following ICV administration of NA, it is virtually absent at the moment when the behavioral evaluations were carried out here, i.e. 2 and 10 weeks post-ICV injection, thus confirming that the resolution of the inflammatory process was progressing as expected^46^.

**Figure 7:**
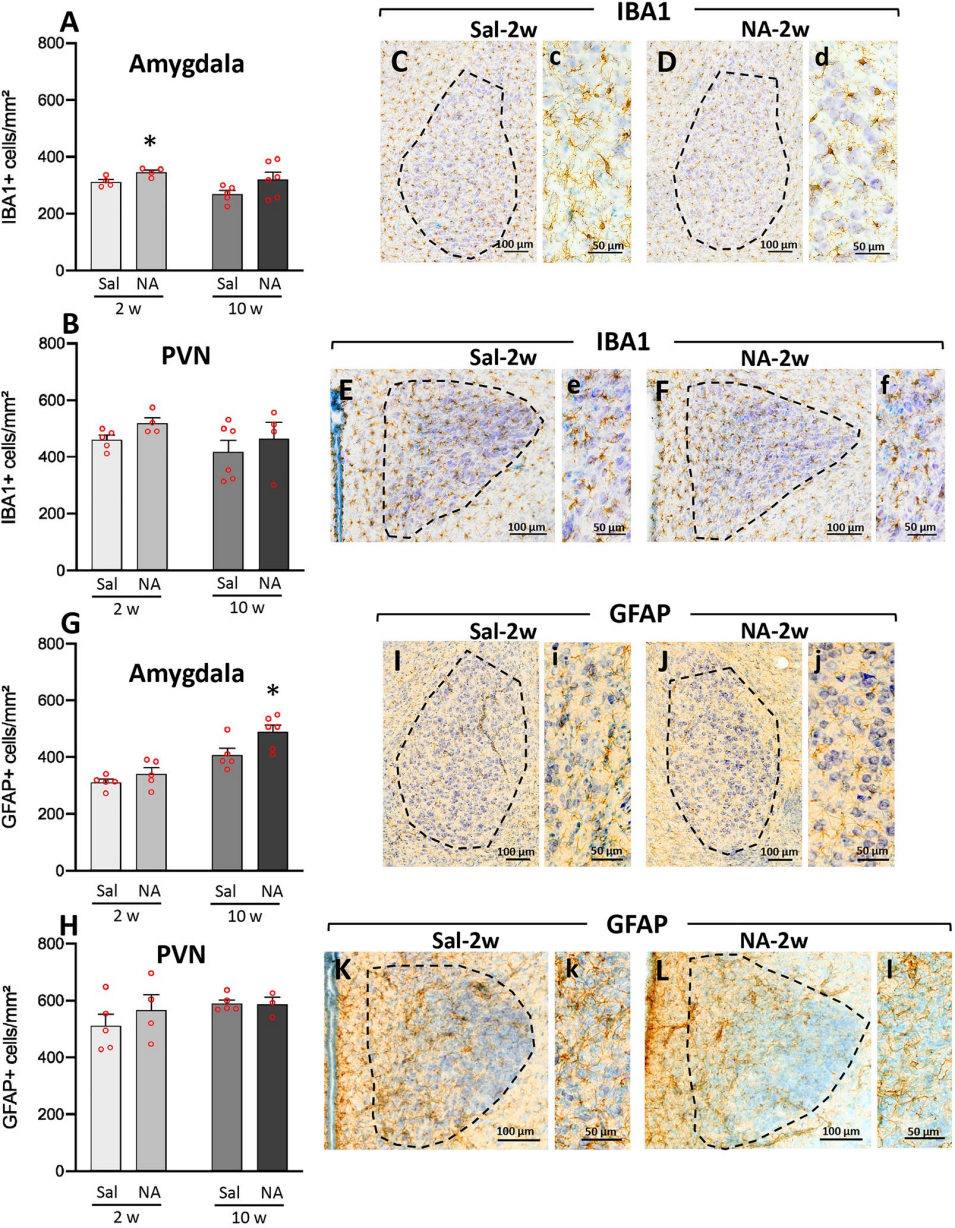
IBA1-positive and GFAP-positive cell counts in amygdala and hypothalamic PVN after neuraminidase induced neuroinflammation. Rats ICV-injected with saline (Sal) or with neuraminidase (NA) were sacrificed 2 weeks (2w) or 10 weeks (10w) after the ICV. Their brains were removed and processed for immunostaining with IBA1 (a widely used marker of microglial cells) or GFAP (a marker of astrocytes). Positive cells are labeled in brown; sections were counterstained with toluidine blue. IBA1-positive cell counts were carried out in two regions related to anxiety and to the stress response: the amygdala (**A**) and the hypothalamic paraventricular nucleus (PVN; **B**). In the same way, GFAP-positive cell counts were performed in the amygdala (**G**) and the PVN (**H**). The bars in histograms represent the mean ± SEM of n = 5–6 animals per experimental group. Student’s t-test were performed in order to check the influence of treatment (Sal versus NA) at both time assessments on the number IBA1-positive or GFAP-positive cells (*p < 0.05). Representative images of IBA-1 labeled amygdala (**C**,**D**) and PVN (**E**,**F**) are shown, as well as GFAP labeled amygdala (**I**,**J**) and PVN (**K**,**L**), all from animals sacrificed 2 weeks after the injection; the area of interest where the cell count was performed is delimited with a broken line. Images **c**–**f** (on the right of **C**–**F**) and **i**–**l** (on the right of **I**–**L**) are a magnified detail of each respective photograph.

The inflammatory status was further assessed by studying resident cells such as microglia and astrocytes. Cell counts were performed in two areas related to stress response and anxiety, namely the amygdala (Fig. 7A,C,D,G,I,J) and the paraventricular nucleus of the hypothalamus (PVN) (Fig. 7B,E,F,H,K,L). In the amygdala, the number of IBA1-positive cells, mostly corresponding to microglia, showed a mild increase 2 weeks after the ICV injection with NA (t = − 2.767, df = 6, *p* = 0.033; Fig. 7A). In spite of a similar tendency, no statistically significant difference was found 10 weeks post-ICV (t = − 1.741, df = 9, *p* = 0.116). Moreover, no significant differences in IBA1-positive cell counts were detected in the PVN neither 2 weeks nor 10 weeks after the ICV (for 2 weeks: t = − 2.304, df = 7, *p* = 0.055; for 10 weeks: t = − 0.690, df = 8, *p* = 0.510; Fig. 7B). However, a slight tendency of IBA1-positive cells to be increased in NA-treated animals was observed (see *p* value at 2 weeks is *p* = 0.055, on the brink of statistical significance), in accordance with cell counts performed in other studies by our group (not published).

Regarding GFAP-positive cells in the amygdala, a statistically significant increase in the number of astrocytes was found 10 weeks after the ICV injection of NA (t = − 2.431, df = 9, *p* = 0.038; Fig. 7G). However, no difference between NA and saline injected rats was observed 2 weeks after the ICV injection (t = − 1.228, df = 8, *p* = 0.254). In the PVN, no differences were detected 2 weeks after the ICV injection (t = − 0.832, df = 7, *p* = 0.433), nor after 10 weeks (t = 0.782, df = 7, *p* = 0.460; Fig. 7H).

In summary, 2 weeks after the ICV injection of NA the number of microglial cells in the amygdala was increased in NA-treated animals, although in the PVN was unchanged. The number of astrocytes, both in the amygdala and the PVN, did not significantly change. However, 10 weeks after the injection an increase in the number of astrocytes was observed in the amygdala, but not in the PVN, of NA-treated rats compared to saline-injected controls.

**Figure 8:**
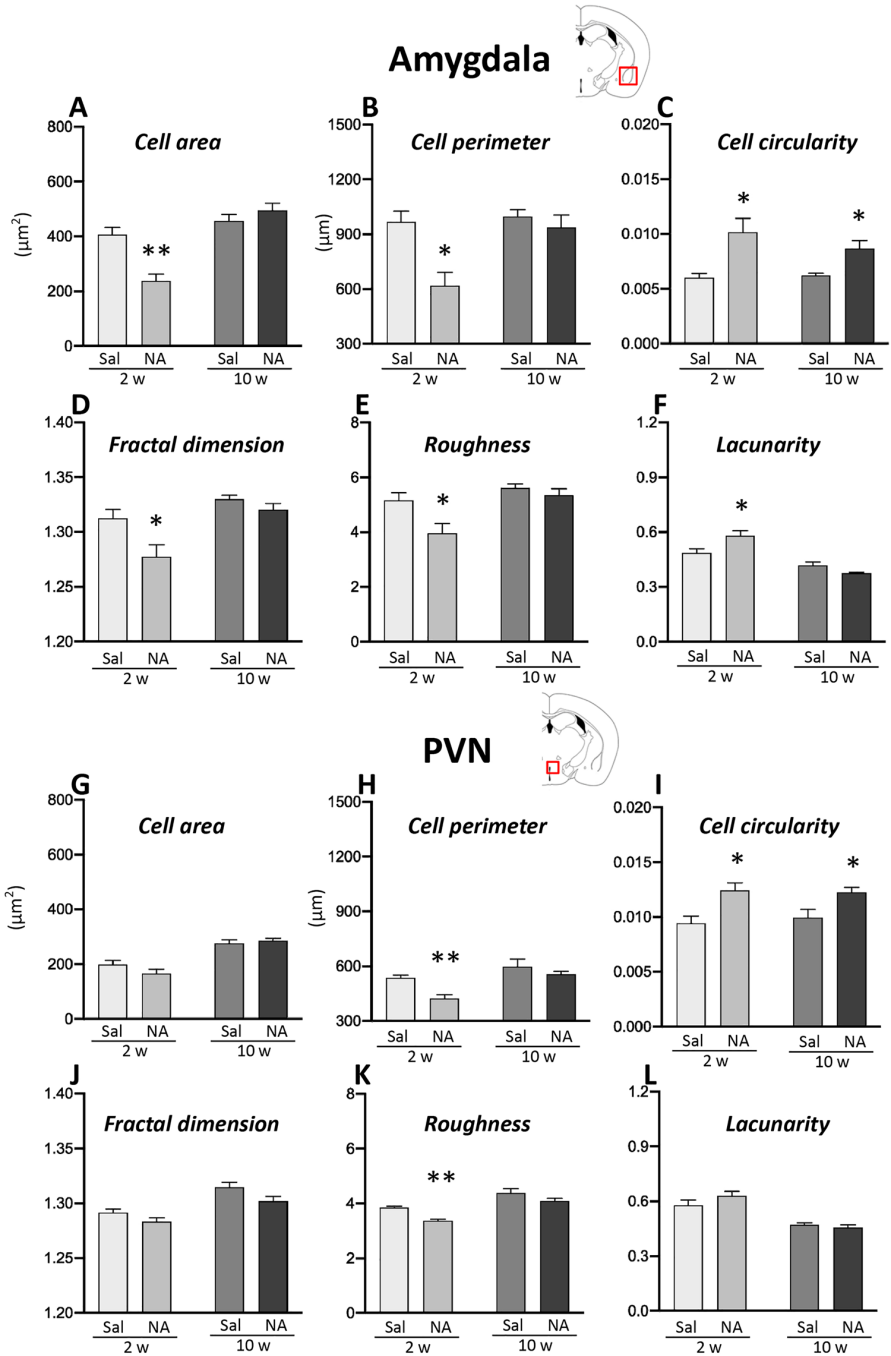
Morphological parameters of microglial cells sampled from the amygdala and PVN of rats subjected to NA-induced neuroinflammation. Rats ICV-injected with saline (Sal) or with neuraminidase (NA) were sacrificed 2 weeks (2w) or 10 weeks (10w) after the ICV injection. Histological sections obtained from their brains were immunostained for IBA1. Images of individual microglial cells located in the amygdala or the PVN were processed to measure morphological parameters. Student’s t-test was performed in order to check the influence of treatment (Sal versus NA) on microglia morphology sampled from different experimental groups at both time points. Here are shown only some of the parameters measured (other parameters available in Supplementary material S1): *cell area* (**A**,**G**), *cell perimeter* (**B**,**H**), *cell circularity* (**C**,**I**), *fractal dimension* (**D**,**J**), *roughness* (**E**,**K**) and *lacunarity* (**F**,**L**). The bars in histograms represent the mean value ± SEM of n = 50–57 cells for amygdala (**A**–**F**) and n = 40–60 cells for PVN (**G**–**L**) per experimental group. Morphological differences between NA and saline treated groups are more evident in amygdala 2 weeks after the ICV-injection, while after 10 weeks most parameters (except for C, *cell circularity*) are similar in both groups. In PVN differences between NA and saline groups are scarce (see **H**,**I** and **K**, *cell perimeter*, *cell circularity* and *roughness*). *p < 0.05; **p < 0.005.

Microglial activation is a hallmark of neuroinflammation, not easily detected if moderate. An alternative approach, objective and quite sensitive, to assess microglial activation is to measure morphological parameters of individual microglial cells^43,55^. This approach was applied to microglial cells sampled from the amygdala and from the PVN. A total of 15 different morphological parameters were measured in these cells (Supplementary material, Fig. S1). Student’s *t*-tests performed with parameters measured in microglia sampled from amygdala (Fig. 8A–F) and from PVN (Fig. 8G–L) showed significant differences in several parameters (results available at Supplementary material, Figs. S3 and S4); only six of them are shown here for simplicity.

Two weeks after the ICV injection, microglial cells sampled from the amygdala of NA-treated rats presented reduced *cell area* (t = 4.568, df = 8, *p* = 0.002; Fig. 8A), *cell perimeter* (t = 3.654, df = 8, *p* = 0.006; Fig. 8B), *fractal dimension* (t = 2.579, df = 7, *p* = 0.037; Fig. 8D) and *roughness* (t = 2.702; df = 8, *p* = 0.027; Fig. 8E), and increased *cell circularity* (t = − 3.435, df = 7, *p* = 0.011; Fig. 8C) and *lacunarity* (t = − 2.702, df = 8, *p* = 0.027; Fig. 8F), compared to cells from saline-treated rats. The same study performed in cells sampled from rats sacrificed 10 weeks after the ICV injection yielded no differences in most of these parameters in NA-treated microglial cells compare to those in saline controls, except for an increase in *cell circularity* (t = − 3.101, df = 8, *p* = 0.015; Fig. 8C). When analyzing morphological parameters of microglial cells located in hypothalamic PVN, most parameters were similar in cells sampled from NA or saline treated rats, except for an increased *cell circularity* in microglia from NA-treated rats compare to saline controls, both 2 (t = − 2.946, df = 6, *p* = 0.026) and 10 weeks (t = − 2.651, df = 8, *p* = 0.029) after the ICV injection (Fig. 8I). *Cell perimeter* and *roughness* were also decreased in microglia from NA-treated rats, but only 2 weeks post-ICV (t = 4.413, df = 6, *p* = 0.005; t = 5.187, df = 6, *p* = 0.002; Fig. 8H,K).

In summary, in NA-treated rats the morphology of microglial cells residing in the amygdala is altered 2 weeks after the injection, indicating a morphological change that is compatible with an activated state. Ten weeks after the injection, such morphological change is mostly reversed. Microglial cells located in the PVN do not seem to suffer such pronounced morphological alteration upon NA treatment, although some parameters (e.g. *cell perimeter, cell circularity* and *roughness*) do in fact change indicating a bias to an activated profile.

## Discussion

Neurological complications or behavioral alterations have been associated to neuroinflammation^19,20^. While some of these symptoms decline with time along with inflammation, the possibility of long-term sequelae needs to be considered. The main goal of this research was to explore if NA-induced neuroinflammation provokes behavioral or neurological disturbances, in particular at medium (2 weeks) and long (10 weeks) terms, aiming to the possibility of long-lasting underestimated sequelae. For this purpose, we first carried out a neurological examination of the animals using a neurological test battery to explore sensorimotor reflexes. Then, the exploratory behavior and the anxiety-like response were examined using the OF test.

Concerning neurological reflexes, a neurological test battery was used to explore whether NA-injection affected a particular brain region, interfered with a specific function, or affected the CNS as a whole^56^. Normal reactions were observed in all neurological reflexes examined, indicating the absence of impairments at any of the post-injection times evaluated. These data suggest that a single ICV dose of NA does not produce neurological sequelae, at least 2 weeks after its administration and onward. The possibility of symptoms at shorter post-injection times cannot be discarded.

On the other hand, no deficits in OF exploration were observed in NA-treated rats, which presented normal locomotor capacity (*distance travelled* and *locomotion speed*) compared to sham animals. Moreover, no differences were observed in the number of entries or in the time spent in the center of maze 2 weeks after NA administration. Surprisingly, an increase in the time spent in the center of the arena was observed in the evaluation performed 10 weeks post-NA injection.

In addition, and based on an approach that relies on monitoring freely behavior of rats^53^, the ethological analysis of particular behaviors exhibited by the animals during the OF test was carried out. Two weeks after NA administration rats showed a reduced time and frequency of *unsupported rearing*, with increased latency to the first episode of this behavior. Nevertheless, no changes were observed between treatments in *supported rearing*. The reduction of *unsupported rearing* behavior does not seem to be related to disturbances in motor functions since neither the *rearing with support* nor the motor parameters assessed in the OF or the sensorimotor reflexes were altered. The impairment observed in *unsupported rearing* might indicate changes in emotional variables. In fact, previous works revealed that separating and independently measuring *supported* and *unsupported rearing* can improve the assessment of the animal’s emotional state^57^, *Unsupported rearing* has been associated with emotionality, whereas *supported rearing* has been preferentially associated with motor variables^58,59^. On the other hand, animals treated with NA showed increased *self-grooming* behavior, often observed in animal models of stress and anxiety^60^. With exceptions, the time spent *grooming* is a well-established indicator of increased anxiety^61–63^, particularly when accompanied by an increased frequency of *grooming* episodes^64^. Here, rats treated with NA increased both the frequency and the duration of *grooming* episodes (and the ratio duration/frequency) 2 weeks after ICV-injection but not later. All these data indicate that NA injected ICV provokes anxiety-like behavior, at least 2 weeks after its administration, an observation that was further confirmed by PCA. This analysis revealed two factors: Factor 1, which grouped motor parameters, and Factor 2, which grouped anxiety-related behaviors. While NA-treated rats did not present differences compared to sham controls regarding Factor 1, conversely an increase in score was observed in Factor 2. Also, in accordance to our above exposed results, these alterations were no longer observed 10 weeks after NA injection.

However, this anxiety-like pattern shown by NA-treated rats is apparently in conflict with the time they spent in the center of the maze. One of the most common behaviors evaluated in the OF is the time that animals spend in the central area of the maze, a parameter that is inversely related to anxiety. The center of the maze is considered to be an aversive zone from which the most anxious animals tend to escape quickly. Thus, the more anxious the animal is, the more time it will spend in the outer area of the arena^65^. Here, in spite of NA-injection inducing an anxiety-like behavior, no difference with sham animals was observed regarding arena zone preference. However, a careful analysis revealed an increase in *freezing* behavior 2 weeks after NA. *Freezing* is defined as the complete absence of body movements except for those of ventilation. This behavior is usually associated with anxiety, with more anxious rats showing longer *freezing* times^66^. As the animals were placed in the center of the arena when starting the OF test, increased *freezing* behavior may have prevented them from escaping from the center of the maze, what could explain the absence of difference between treatments in the time spent in this area. Unexpectedly, 10 weeks post-injection NA-treated animals stayed longer in the center of the maze. Although this might suggest an anxiolytic effect of NA in the long term, it is more likely to be due to impairment in long-term habituation. Impaired habituation to the OF may reflect recognition memory problems, which could be a long-term consequence of NA-injection. Similar results have been observed 12 weeks after the administration of LPS, a widely used neuroinflammatory agent: LPS-treated mice exhibited increased distance traveled in the OF arena^67^. Future studies are needed to explore the long-term effects of NA, primarily on recognition memory.

Taken together these data indicate that a single ICV injection of NA induces increased anxiety-like behavior without provoking, however, neurological or motor disturbances. Such symptoms are observed 2 weeks after the injection, but not at longer times (10 weeks post-injection). In the long term, NA may induce problems in recognition memory, a possibility that needs to be explored.

Several pieces of evidence show that continued low-grade inflammation is a risk factor for neuropsychiatric disorders, including anxiety disorders^68,69^. In this sense, it has been reported that increased infiltration of peripheral monocytes may influence anxiety-like behavior^70^. In addition, low-grade of inflammation is present in anxiety disorders^71^ particularly in brain regions critical for the regulation of anxiety^72^. In search for signs of neuroinflammation which could be associated to the anxiety-like behavior detected 2 weeks after NA administration, no peripheral cell infiltration was observed at the times post-injection studied, which is in accordance with previous studies using this model^46^. Subsequently, and given that hypothalamic inflammation has been associated with the onset of signs of anxiety^73^, the mRNA levels of various genes related to inflammation were measured in the hypothalamus by qPCR. The hypothalamus is part of the circuitry that mediates many innate behaviors, among them the self-grooming triggered by anxiety^74^. In fact, this brain region has been recently implicated in the development of pathological grooming^75–77^ and in changes in rearing behavior due to anxiety^75^. Indeed, it has been demonstrated that specific activation of corticotropin-releasing hormone (CRH) in PVN is sufficient to increase grooming and decrease rearing^75^. Moreover, the hypothalamus is involved in autonomic response to stress^78,79^. In the present work, an overexpression of some genes related to inflammation (the pattern recognition receptor TLR4 and the alarmin HMGB1) was found in the hypothalamus of NA treated rats concurrently with the anxiety-like behavior (i.e. at 2 weeks but not at 10 weeks). The overexpression of both genes may be linked to the increased anxiety-like behavior. In this sense, systemic anti-HMGB1 antibody treatment exerted neuroprotective effects and improved anxiety in aged rats^80^. However, mice treated with the HMGB1 inhibitor glycyrrhizin had an increased post-stroke anxiety-like behavior^81^. On the other hand, TLR4 plays an essential role in the regulation of functional emotional response, the dysregulation of which induces anxiety-like behavior^82^. Both and increase in anxiety after stimulation with LPS^83^ and an anxiolytic effect after pharmacological blockade of TLR4, have been observed^84^. Overall, the observed overexpression of HMGB1 and TLR4 could be mediating the anxiety-like behavior observed 2 weeks after NA injection. By contrast, the increased mRNA levels of NLRP3 and TNFα observed at 10 weeks post-ICV were not apparently related to emotional behavioral changes. However, such long lasting overexpression of these inflammation related genes might represent an evidence of a mild inflammation, which could be worth investigating, as mild inflammation has been related to behavioral disturbances^85^.

Together with the hypothalamus, the amygdala, a limbic brain structure that is involved in the regulation of motivational states, has a crucial role in the modulation of anxiety^86^. The amygdala, which is also involved in self-grooming^87^, projects to respective divisions of the bed nucleus of the stria terminalis (BNST), the main connector between the amygdala and the hypothalamus^88,89^, and to the medial hypothalamus^90^. Although the amygdala was not initially included as target region for mRNA expression studies in the present work, the behavioral results observed prompted us to explore the inflammatory status of both amygdala and hypothalamus, in this case by studying, in histology sections, resident cells involved in the immune response, such as microglia and astrocytes. The histological study revealed microgliosis in amygdala (and a similar tendency in PVN) 2 weeks after NA administration, which was not maintained after 10 weeks. Also, astrogliosis in amygdala was observed, but only at long term (10 weeks) after ICV-NA. These results pointed to mild gliosis in these structures induced by NA injection, particularly in amygdala. Moreover, the morphological analysis of microglial cells, a quite sensitive tool to evaluate cell activation,  demonstrated that, in the amygdala of NA injected rats, these cells presented a morphology consistent with an activated state (decreased *cell area, cell perimeter, fractal dimension* and *roughness*; increased *cell circularity* and *lacunarity*) compared with those cells from control animals^43,55^. This morphological change observed in the microglial population of the amygdala 2 weeks after the administration of NA was virtually reverted 10 weeks later, what supports the possibility that microglia activation in amygdala might be related to the NA-induced anxiety-like behavior detected. The same measurements performed in microglia located in the PVN yielded a similar tendency (i.e. a more activated profile in cells from NA treated rats), but for most morphological parameters the differences were not statistically significant. Unfortunately, samples of amygdala for gene expression studies were not obtained.

In accordance with our results, it was reported increased microglial activation in the amygdala and anxiety-like behavior in mice after the intraperitoneal injection of LPS^20^. In addition, fluoxetine pretreatment significantly prevented LPS-induced anxiety-like behavior, which was accompanied by reduced activation of microglia^20^. However, in this work behavioral evaluation was performed in the acute phase of inflammation (24 h after the LPS challenge). Along the same lines, an association of repeated social defeat stress-induced microglial activation in amygdala with anxiety-related behaviors has been observed in adult male rats^91^. In relation to the hypothalamus, although it is a crucial structure for the endocrine response to stress, to our knowledge there are no studies linking hypothalamic microglial activation to the onset of anxiety. Our data reveal subtle morphological changes of microglia located in the PVN after NA-injection, which suggest a mild activation of these cells, but in no case to the extent observed in microglia located in the amygdala, which seems to be much more sensitive to the inflammatory stimulus applied. Further studies are necessary to determine if there is any relationship between hypothalamic microglia and anxiety symptoms.

Altogether, these results point out that NA administered ICV may cause anxiety-like behavior in the medium term, while not affecting other functions like sensorimotor functions or the locomotor capacity. Such behavioral alteration is transient, as it is not detected later. The anxiety-like behavior concurs in time with a mild inflammation, evidenced by the overexpression of certain genes and, more notably, by the morphological bias of microglial cells towards an activated profile, particularly in the amygdala, what could represent a histological support of the anxiety-like behavior observed.

On the other hand, and although it is not the focus of the present study, it is worth noting that chronic neuroinflammation can lead to behavioral and cognitive alterations, or even major neurological problems. Key molecules in such processes, implicated in various neuropathological conditions, are the intracellular multi-protein complexes inflammasomes. Dysregulation of the inflammasome NLRP3 has been associated with the onset and progression of a wide range of neurological conditions such as Alzheimer’s and Parkinson’s diseases, among others^92–94^. Our results showed increased mRNA levels of the inflammasome protein NLRP3 in the hypothalamus long after the injection of NA. Future studies should explore whether such increased expression is sustained over time and if it might be related to the development of cognitive impairment (for instance, on recognition memory, as some evidences reported here suggest its possible alteration) and neurodegenerative diseases.

Finally, another aspect worth to be considered for future studies is the possibility that a single neuroinflammatory event could induce immune memory in the brain^35,95^. Immune training results in enhanced immune responses upon a second challenge, which could also have behavioral consequences. Primed microglia are proposed to be responsible for exacerbated neuroinflammatory responses^4,96,97^. Evidences of primed hypothalamic microglia three months after NA-induced inflammation have been recently reported. Moreover, this fact has been related to altered hypothalamic functions, such as energy balance regulation^98^. Therefore, a paradigm where a second stimulus is applied long after NA injection might eventually highlight behavioral disturbances.

## Conclusion

In this work we demonstrate for the first time that the intraventricular administration of NA, a sialidase enzyme beared by several pathogens including influenza virus, provokes increased anxiety-like behavior without inducing neurological impairments. These effects, which are observed 2 weeks after NA injection (long after the acute phase of inflammation) but disappear 10 weeks later, have been linked to mild inflammation, which particularly affects the amygdala. However, future studies should aim to find out if NA injection (or brain infections caused by NA bearing microbes such as influenza, measles or mumps) may trigger anxiety disorders or other behavioral disturbances in the long term, or if those disorders may become chronic over time. These results are relevant in view of the recurrence of viral infections, and may help to the development of more effective treatments for anxiety disorders with low levels of underlying neuroinflammation.

## Supplementary Information

Supplementary Information.

## Data Availability

The datasets generated during and/or analyzed during the current study are available from the corresponding author on request.
