# Supplementary material for: Anxiety-like behavior and microglial activation in the amygdala after acute neuroinflammation induced by microbial neuraminidase
Source: Sci Rep. 2022 Jul 8;12:11581. doi: 10.1038/s41598-022-15617-5 (PMC9270343; doi:10.1038/s41598-022-15617-5)
Supplement: Supplementary file 1 — Supplementary Information. [file 41598_2022_15617_MOESM1_ESM.pdf]

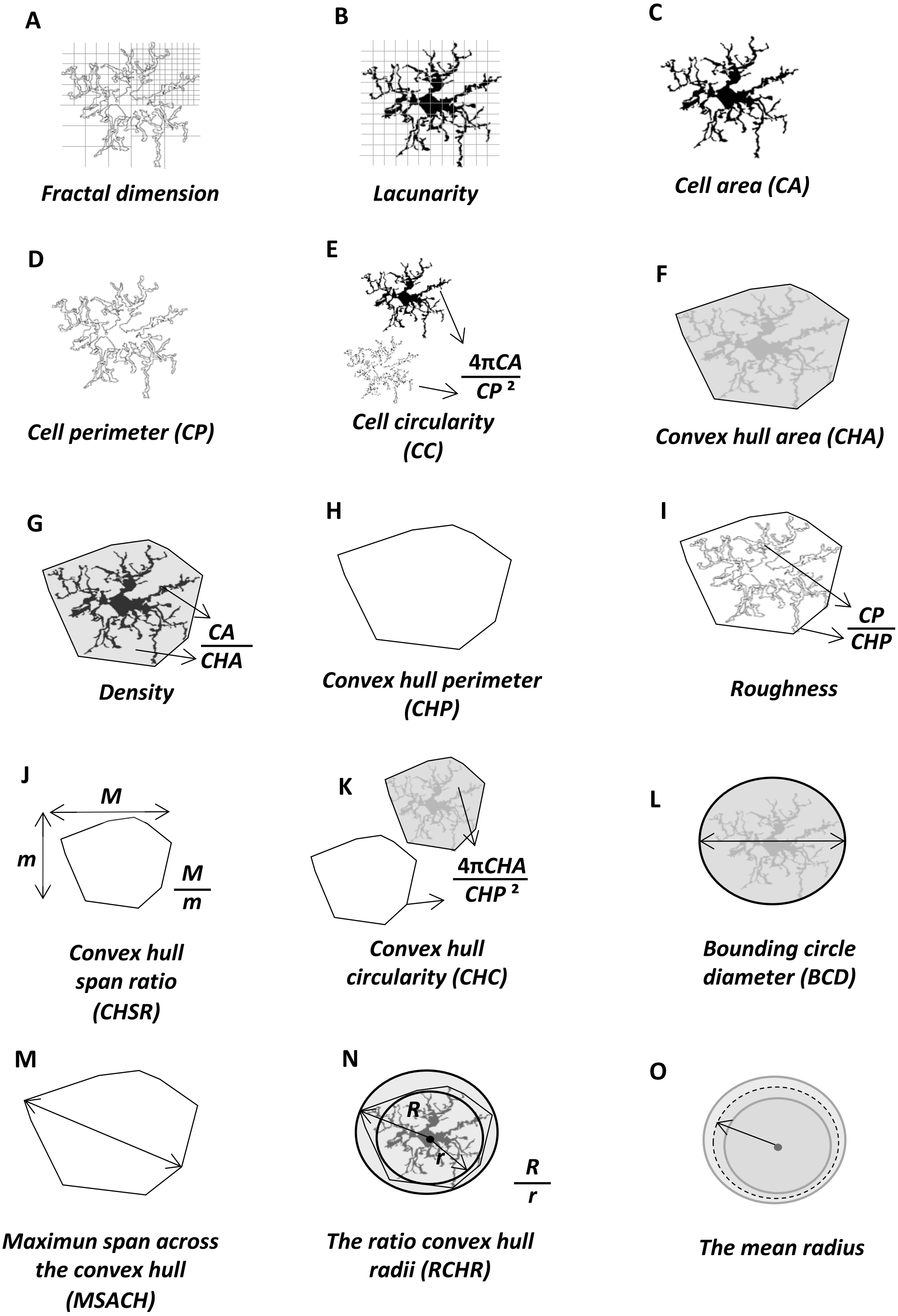

Fig. S1. A schematic explanation of the morphological parameters measured in individual microglial cells. A comprehensive explanation may be found in Fernández-Arjona et al., 2017.
